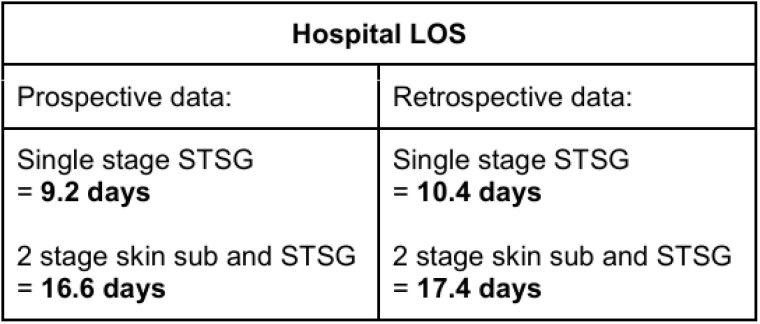# 691 Effectiveness of an Early Mobility Timeline for Patients with Lower Extremity Split Thickness Skin Grafts

**DOI:** 10.1093/jbcr/iraf019.320

**Published:** 2025-04-01

**Authors:** Connie Yeung

**Affiliations:** Bothin Burn Center

## Abstract

**Introduction:**

The long-standing mobility guideline for patients with lower extremity (LE) split-thickness skin grafts (STSG) in our burn unit is as follows: post-op day (POD) #1-4 bed rest, POD#5 edge of bed sitting, POD#6 transfers, POD#7 ambulation. Prolonged bed rest, however, can result in detrimental physiological effects, and practice guidelines for early ambulation have recommended mobility earlier than POD#5 for burn patients with LE STSG. Therefore, an early mobility timeline was developed to mobilize patients with LE STSG on POD#3. We hypothesize that early mobility will not result in graft loss when external compression is utilized, and hospital length of stay (LOS) will decrease.

**Methods:**

The developed early mobility timeline is as follows:

POD#3: transfer out of bed to a chair with LE ace wraps; use splints when STSG crosses a joint

POD#4: remove splints, ambulate in-room distances

POD#5: ambulate hallway distances

A prospective case series study was completed from October 2021 to April 2023 (1.5 years), during which 51 patients with LE STSG received early mobility. Any graft loss on the day of dressing takedown (POD#4) and hospital LOS were recorded. Patients who received a single-stage STSG or a two-stage skin substitute followed by STSG surgeries were included.

A retrospective data collection of the 1.5 years before the initiation of early mobility was also performed. 32 patients were identified; all had mobilized on POD#5 and after. This was used to compare the hospital LOS before and after implementing early mobility for patients with LE STSG.

**Results:**

On graft loss:

51 patients who received early mobility had TBSA involvement between 1-25% (avg 6.8%). 49 of them did not demonstrate LE graft loss during dressing takedown. Of the 2 patients that did present with graft loss, both graft losses were < 1% TBSA and healed without further surgery. No other complication occurred during early mobility intervention.

On hospital LOS:

Hospital LOS was compared between the prospective and retrospective groups. In the retrospective group, 32 patients who were mobilized on POD#5 and after had TBSA involvement between 3-45% (avg 11.3%). When early mobility was utilized in the single-stage STSG group, LOS decreased by 1.2 days. In the 2-stage skin substitute followed by the STSG group, LOS decreased by 0.8 days.

**Conclusions:**

Early mobilization for patients with LE STSG can be successfully implemented on POD#3 with external compression. Most patients (96%) did not demonstrate graft loss, and the minority of patients who had minimal graft loss healed without further surgery. Hospital LOS also decreased by an average of 1 day when patients were mobilized on POD#3 instead of POD#5.

**Applicability of Research to Practice:**

Early mobility for patients with LE STSG can be safely implemented and help to decrease hospital LOS. Increased research on the success of early mobility programs can contribute to the possible standardization of mobility protocols within burn rehabilitation.

**Funding for the Study:**

N/A